# Isolation, Characterization, and Screening of Antimicrobial-Producing Actinomycetes from Soil Samples

**DOI:** 10.1155/2020/2716584

**Published:** 2020-03-26

**Authors:** Anupama Sapkota, Aishwarya Thapa, Anupa Budhathoki, Muskan Sainju, Prativa Shrestha, Sagar Aryal

**Affiliations:** ^1^Department of Microbiology, St. Xavier's College, Maitighar, Kathmandu, Nepal; ^2^Department of Natural Products, Kathmandu Research Institute for Biological Sciences, Lalitpur, Nepal

## Abstract

Actinomycetes are Gram-positive, facultative anaerobic fungus-like filamentous bacteria which remain on the top of the natural antibiotic producers. Due to the climatic and geographical diversity of Nepal, a wide range of microorganisms with potent source of antimicrobials are available. The objective of this study was to isolate, identify, and screen the potential antimicrobial-producing actinomycetes from soils covering different altitude range of Nepal. Forty-one isolates of actinomycetes were isolated from 11 soil samples collected from different locations in Nepal with altitude ranging from 1500 to 4380 meters. The isolates were identified on the basis of morphological study, different sugar utilization, protein utilization, and hydrolysis tests. They were also characterized on the basis of temperature and pH. Primary screening for antimicrobial activity was carried out against several test organisms: *Staphylococcus aureus* (ATCC 25923), *Escherichia coli* (ATCC 25922), *Klebsiella pneumoniae* (ATCC 700603), and *Pseudomonas aeruginosa* (ATCC 27853) by the perpendicular streaking method, and secondary screening was carried out by the agar well diffusion method using ethyl acetate for solvent extraction. 70.7% of the isolates were identified as *Streptomyces* spp., 19.5% as *Nocardia* spp., and 9.5% as *Micromonospora* spp. 43.34% of actinomycete isolates was found to be potent antimicrobial producers from the primary screening among which 46.34% were effective against Gram-positive and 12.19% against Gram-negative test organisms. Isolate C7 (*Micromonospora* spp.) showed the best broad-spectrum antimicrobial activity during secondary screening. A total of 11 different types of pigments were observed to be produced by different isolates, of which, the yellow pigment was the most prominent. The association between elevation, pH, and pigment with the antimicrobial production was found to be insignificant. This finding can be of importance for further investigation towards obtaining broad-spectrum antibiotics for therapeutic purpose.

## 1. Introduction

Actinomycetes are the heterogeneous group of microorganisms, which have thread-like filaments in the soil [1]. They are extensively distributed in natural habitat and entailed in different biological and metabolic processes, for instance, useful for producing extracellular enzymes [2]. In addition, almost 90% of actinomycetes genera have been isolated from soil, which are innocuous for different fields: industrial and pharmaceutical sectors [3]. Besides, actinomycetes produce instinctive pigments on the media which are red, green, yellow, brown, and black in color [4]. It has been posited that *Streptomyces* are putative organisms that encompasses the production of antibiotics followed by *Nocardia* and *Micromonospora*, which are inferior in nature when compared to *Streptomyces* in the ability to produce antibiotics [5].

Researchers have done many studies on isolation and screening of antimicrobial-producing actinomycetes. It has propounded that most of the novel antibiotics have been found by screening of isolates from soil [6]. However, due to the emergence of multidrug-resistant pathogens, antimicrobial resistant is on the rise, which is pernicious to the health of the large population of community. Moreover, it has been a serious problem in the treatment of infectious disease, so, the expatiating on this topic is needed to discover new novel antibiotics that help in controlling the problem [7, 8].

As we know, Nepal is a geographically diverse country which is divided into three regions: the mountain region, the hilly region, and the terai region. Based on their variation in altitude and soil type and their contents, there is a possibility of observing similar microflora, which conjectures to vary the distribution of antimicrobial-producing actinomycetes [9]. So, the present study was done to isolate and screen the antimicrobial-producing actinomycetes from soil samples, which were collected from different locations with varying altitudes starting from 1500 masl to 4380 masl of Nepal. Thus, this project helps in analyzing and reporting distribution of the antimicrobial activities of microorganisms collected from sample sites.

## 2. Materials and Methods

### 2.1. Soil Sample Collection

Eleven soil samples from different altitude range of Nepal (organically cultivated fields, rhizospheric area, and river banks) were processed during the research. The samples were named with alphabet A, B, and so on with increasing order of their altitude. Similarly, the isolates from each respective sample were labeled as A1, A2, and so on. The soil was collected within the depth of 10–12.5 cm. The samples were collected in sterile polythene bags, closely tightened, and were taken to the laboratory.

### 2.2. Isolation of Actinomycetes

One gram of soil sample was taken and serially diluted up to 10^−2^ using distilled water as a diluent. The mixture was shaken vigorously using a vortex; 0.1 ml of each dilution was placed on starch casein agar (composition: soluble starch: 10 g, K_2_HPO_4_: 2 g, KNO_3_: 2 g, casein: 0.3 g, MgSO_4_.7H_2_O: 0.05 g, CaCO_3_: 0.02 g, FeSO_4_.7H_2_O: 0.01 g, agar: 15 g, and filtered sea water: 1000 ml and pH: 7.0 ± 0.1), and the inoculum was spread properly using a sterile glass spreader. The inoculated plates were allowed to stand at room temperature for 5–10 minutes to allow the liquid to be absorbed and were incubated at 28°C for 7 days.

### 2.3. Identification

Identification of the actinomycetes was done on the basis of macroscopic and microscopic examination and physiological tests as suggested by Bergey's Manual of Systematic Bacteriology, 2^nd^ Edition, Vol 5, The Actinobacteria, Part A.

### 2.4. Macroscopic Characterization

The isolated actinomycetes were observed for aerial mycelium, submerged mycelium, color, and diffusible pigments.

### 2.5. Microscopic Observation

Microscopic examination was performed by cover slip and Gram-staining method. Sterile cover slip was inserted into the solidified starch casein agar medium plate at an inclination of 45° with the agar surface. Actinomycete isolate was inoculated along the surface of the medium that meets the surface of the buried cover slip. It was incubated at 28°C for four days. The cover slip was removed using sterile forceps and placed on an individual clean glass slide, which were then observed at oil immersion objective [10].

### 2.6. Biochemical Tests

For all the isolates, a loopful of colony of pure culture of about 7 days of incubation was placed in starch casein broth and incubated at 28°C for 4 days. After the appearance of turbidity, the culture suspension was used for different sugar utilization tests, protein utilization test, and catalase and oxidase tests. Different hydrolysis tests, namely, starch hydrolysis, casein hydrolysis, lipid hydrolysis, and gelatin hydrolysis tests, were performed.

## 3. Primary Screening

Primary screening of actinomycetes was performed on the Mueller–Hinton agar medium employing the perpendicular streak method. In the sterile agar medium, the pure isolate of actinomycetes was streaked along the diameter of the plate. The plate was incubated at 28°C for 7 days. Pure colony of test bacteria *Staphylococcus aureus* (ATCC 25923), *E. coli* (ATCC 25922), *Pseudomonas aeruginosa* (ATCC 27853), and *Klebsiella pneumoniae* (ATCC 700603) was transferred into fresh nutrient broth and incubated at 37°C for 24 hours until the visible turbidity. After adjusting the turbidity equal to that of 0.5 McFarland with the cell count of 1.5 × 10^8^, the test organisms were streaked perpendicular to the isolate. The plates were further incubated at 37°C for 24 hours, and the antimicrobial activity was estimated from the zone of inhibition of test organism [11].

## 4. Secondary Screening

### 4.1. Production of Crude Extract

Based on the results of primary screening, each isolate was subjected to submerged fermentation in the ISP2 medium (composition: yeast extract: 4 g, malt extract: 10 g, dextrose: 4 g, agar: 15 g, and filtered sea water: 1000 ml and pH: 7.3) and Kuster's agar (composition: glycerol: 10 g, casein: 0.3 g, KNO_3_: 2 g, K_2_HPO_4_: 2 g, soluble starch: 0.5 g, asparagine: 0.1 g, FeSO_4_.7H_2_O: 0.01 g, CaCO_3_: 0.02 g, MgSO_4_.7H_2_O: 0.05 g, agar: 15 g, and filtered sea water: 1000 ml and pH: 7.0 ± 0.1) in a shaker incubator at 120 rpm for 7 days at 28°C. The extract was separated by centrifugation at 13000 rpm for 10 minutes at 4°C. The supernatant was mixed with equal volume of ethyl acetate and left overnight. Subsequently, the upper layer of ethyl acetate was again separated and evaporated at 40°C. The concentrated solvent was used for assay [12].

### 4.2. Agar Well Diffusion Method

Five wells of 6 mm diameter were made on the agar plate with the help of sterile cork borers. The test organism was swabbed on the agar surface and 100 *μ*L of crude extract was poured in the wells. Ethyl acetate and antibiotic discs (oxacillin, nitrofurantoin, and ciprofloxacin) were used for negative and positive control, respectively. The plates were allowed to stand for few minutes and incubated at 37°C without inverting for 24 hours.

## 5. Results

In this study, 11 soil samples collected from different locations in Nepal with varying altitude of 1500 to 4380 m. The description of soil samples with the number of isolates is presented in Table 1.

**Figure 1:**
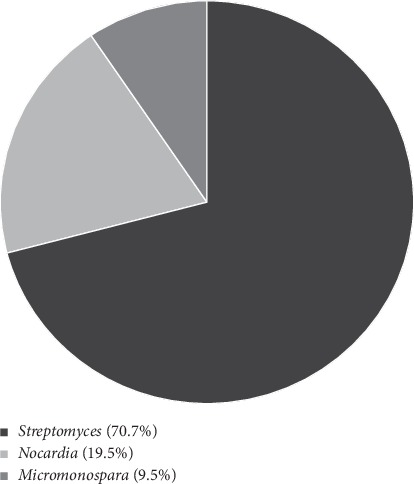
Genera of isolates based on microscopic observation.

A total of 41 isolates were then identified as *Streptomyces* spp. (29 isolates), *Nocardia* spp. (8 isolates), and *Micromonospora* spp. (4 isolates) on the basis of microscopic observation and biochemical tests, as shown in Figure 1 and Table 2.

**Figure 2:**
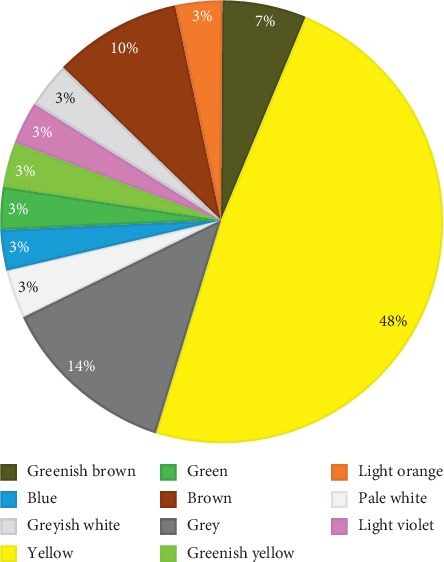
Types of pigment produced by isolates on starch casein agar.

**Figure 3:**
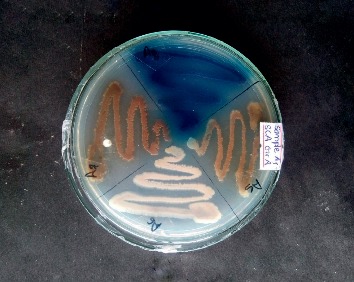
Production of pigment by sample A5, A6, A7, and A8 in the SCA medium.

Most isolates gave different pigmentation, as shown in Figure 2. The color of colonies varied accordingly from yellow (48%), brown (10%), gray (13%), and greenish brown (6%) and 3% had blue, light violet, greenish yellow, blackish grey, grayish white, light pink, and greyish black to white pigments.  Figure 3 shows the production of pigment by A5, A6, A7, and A8 isolates.

The identified isolates were then subjected to primary screening as presented in Table 3. Out of 41 pure isolates, 19 isolates showed antimicrobial activity against the test organisms during primary screening by the perpendicular streaking method. 12.19% of total isolates was found to be active against both Gram-positive and Gram-negative test organisms.

Among 19 active isolates, 13 isolates showed zone of inhibition against the test organisms in the secondary screening, as shown in Table 4. All of the 13 isolates showed activity against *Staphylococcus aureus*, 5 against *Klebsiella pneumoniae*, and 7 against *E. coli*. 7 isolates produced antimicrobial activity against more than one test organism.

## 6. Discussion

The study was carried out to discover the antimicrobial-producing actinomycetes from various geographical regions with altitudinal variation in Nepal. A total of 11 soil samples from locations with altitude ranging from 1500 to 4380 masl were studied. Forty-one actinomycete strains were isolated and identified which were genus *Streptomyces* (70.7%), *Nocardia* (19.5%), and *Micromonospora* (9.5%).

The highest number of actinomycetes was isolated from soil sample collected from Jomsom. The survival of the microorganisms even in former harsh and challenging habitation might be due to their adaptation to the environment and ability to produce resistant structures such as spores [9].

All the isolates were slow-growing, aerobic, glabrous, and folded with diverse colored aerial and substrate mycelia. Most of them produced pigments, namely, brown, yellow, blue, bluish green, and purple, on starch casein agar (SCA). In a study, amongst 7 different media used, the isolate AR-ITM02 (*Streptomyces* spp.) showed pigment-producing ability on SCA only which may be due to enough amount of nutrient available in the media [13]. In another study, 5 different pigments like green, orange, white, yellow, and brown were extracted from actinomycete isolates and were purified and tested for antimicrobial activity which showed that all extracted pigments inhibited growth of *E. coli*, *Staphylococcus aureus*, *Bacillus subtilis*, and *Salmonella typhi* except the yellow pigment which showed inhibitory effect against only Gram-negative bacteria [14].

In the current study, when the total isolates were subjected to primary screening, 46.34% were found to be active against the test organisms. All active isolates showed antibiotic activity against *Staphylococcus aureus* (ATCC 25923), whereas only 5 of them showed activity against *E. coli* (ATCC 25922) and *Klebsiella pneumoniae* (ATCC 700603). None showed activity against *Pseudomonas aeruginosa* (ATCC 27853). In a study, among 117 actinomycete isolates, antibacterial activity was observed in only 12.82% of the isolates [15]. In another study, 6 out of 20 strains showed activity against Gram-positive test organisms and only 4 strains showed activity against Gram-negative organisms [16]. Similarly, out of 106 isolates, only 36 showed activity against test organisms among which 2 were active against only Gram-negative organism, 8 against Gram-positive organisms, and 26 against both Gram-positive and Gram-negative organisms [17].

According to Shirling and Gottlieb [18], the reason for different sensitivity between Gram-positive and Gram-negative bacteria could be explained with respect to the morphological differences between these microorganisms. Gram-negatives have an outer polysaccharide membrane making the cell wall impermeable to lipophilic solutes; however, the Gram-positives have only an outer peptidoglycan layer which is not an effective permeability barrier.

During secondary screening of active isolates using the ethyl acetate extract, only 13 isolates showed zone of inhibition. In a study, the purified ethyl acetate extract of culture broth of *S. sannanensis* SU118 led to the recovery of a potent antibacterial agent active against Gram-positive bacteria [19]. In another study, the ethyl acetate: methanol extract was found to be more effective with antimicrobial activities than those extracted using the water solvent. This might be due to the solubilization of the active compound in the organic solvent compared to the aqueous solvent and also due to the increased concentration of the metabolite in the solvent-extracted mixture [20].

The active isolates showed different activity during primary and secondary screening. According to Bushell et al. [21], during the screening of secondary metabolite, actinomycete isolates which show antibiotic activity on agar but not in liquid culture are often encountered. This might be due to the differences in the substrate composition of the culture media, inoculum size, or incubation time in primary screening and submerged fermentation. It may also be possible that the active compounds released by actinomycetes become inactive or chemically modified or bind to the component of broth. Also, many of the actinomycetes are poor fermenters [9, 22].

Different environmental constrains and growth conditions influence the growth and diversity of actinomycetes, temperature being one of them. It has profound effect in the physiology, morphology, sporulation, biochemistry, and also antimicrobial metabolite production of organisms. In the study, the optimum temperature for the growth of actinomycetes was found to be in the mesophilic range.

In this study, 78.04%, 73.17%, 80.48%, and 41.46% of the isolates produced gelatinase, caseinase, amylase, and lipase, respectively. 78.04% of the isolates were able to show more than one enzymatic activity. In a similar study conducted by Jeffrey [23], approximately 98.4% of the total isolates produced one or more enzymatic activity. The potential of actinomycetes to secrete broad-range enzymes may have resulted from the natural selection in order to survive in a competing environment [24].

## 7. Conclusions

An attempt to isolate different strains of actinomycetes possessing antimicrobial activity from different soil samples of Nepal was made. Forty-one isolates were obtained from 11 soil samples and identified as *Streptomyces* spp. (70.7%), *Micromonospora* spp. (19.5%), and *Nocardia* spp. (9.5%). The best broad-spectrum antibiotic activity from secondary screening was shown by isolate C7. Seven isolates were able to show broad-spectrum antimicrobial activity. At present, there is a need to find out novel antimicrobial-producing strains as the pre-existing drugs have failed due to the development of resistance among the microorganisms. The present study is also a little contribution towards this need. The isolates which showed broad-spectrum activity against the test microorganisms can be considered as candidates in regards of searching for potential antimicrobial compounds.

## Figures and Tables

**Table 1:** Description of soil samples along with the total number of isolates and active isolates.

Soil sample	Altitude (m)	Location	Physical characteristics of soil			No. of actinomycete isolates	No. of active actinomycete isolates
			pH	Color	Consistency		
A	1500	Sundarijal	7.56	Grey	Moist	8	3
B	2000	Kalinchowk	6.97	Brown	Moist	0	0
C	2200	Nagarkot	6.79	Light brown	Moist	4	4
D1	2156	Chandragiri	6.86	Brown	Dry	1	1
D2	2485	Chandragiri	6.72	Brown	Dry	0	0
E	1750	Chitlang	6.51	Black	Dry	8	3
G	3800	Khopra	6.10	Brown	Dry	0	0
H	2743	Jomsom	5.50	Grey	Dry	13	5
I	3500	Tipling	5.54	Brown	Dry	0	0
J	3710	Muktinath	5.73	Grey	Dry	6	3
K	4380	Gosainkunda	5.83	Black	Moist	1	0

**Table 2:** Enzymatic characteristics of the isolates.

Sample	Catalase	Oxidase	Gelatinase	Amylase	Lipase	Caseinase
A1	−	−	+	+	−	−
A3	−	−	−	−	−	−
A4	+	−	−	−	+	−
A5	−	−	+	+	−	+
A6	+	−	−	+	−	+
A7	−	−	+	+	−	+
A8	+	−	+	+	−	+
A11	−	−	−	−	−	−
C1	+	−	−	−	−	+
C2	−	−	−	+	−	−
C7	+	−	+	+	−	+
C8	+	−	+	+	−	−
D1	+	−	+	+	−	+
E1	+	−	+	+	−	+
E3	+	−	+	+	−	+
E4	+	+	−	−	−	−
E5	+	+	+	+	−	−
E7	−	−	+	+	+	−
E8	+	−	+	+	−	+
E14	+	+	+	−	+	−
E15	+	+	+	−	+	+
H1	+	+	+	+	+	+
H2	+	+	+	+	+	+
H3	+	−	+	+	−	+
H4	+	+	+	+	+	+
H5	−	+	+	+	+	+
H6	+	+	+	+	+	+
H7	+	+	+	+	+	+
H11	−	+	+	+	+	+
H12	−	−	+	+	+	+
H13	+	+	+	+	+	+
H15	+	−	+	+	+	+
H16	+	+	+	+	−	+
H17	+	+	+	+	+	+
J1	−	+	−	+	−	+
J4	+	+	+	+	−	+
J6	+	−	+	−	+	+
J8	+	−	+	+	−	+
J10	+	−	+	+	−	+
J12	−	+	+	+	−	+
K1	+	+	−	+	−	+

**Table 3:** Primary screening results of the isolates.

Sample	Identified as	Zone of inhibition (mm)			
		*Staphylococcus aureus* (ATCC 25923)	*E. coli* (ATCC 25922)	*Klebsiella pneumoniae* (ATCC 700603)	*Pseudomonas aeruginosa* (ATCC 27853)
A1	*Nocardia* spp.	0	0	0	0
A3	*Streptomyces* spp.	0	0	0	0
A4	*Streptomyces* spp.	0	0	0	0
A5	*Streptomyces* spp.	0	0	0	0
A6	*Streptomyces* spp.	9	0	0	0
A7	*Streptomyces* spp.	14	0	0	0
A8	*Micromonospora* spp.	8	0	0	0
A11	*Streptomyces* spp.	0	0	0	0
C1	*Streptomyces* spp.	20	19	18	0
C2	*Streptomyces* spp.	30	10	10	0
C7	*Micromonospora* spp.	15	9	12	0
C8	*Streptomyces* spp.	18	15	10	0
D1	*Streptomyces* spp.	3	0	0	0
E1	*Streptomyces* spp.	0	0	0	0
E3	*Nocardia* spp.	0	0	0	0
E4	*Nocardia* spp.	15	10	10	0
E5	*Streptomyces* spp.	0	0	0	0
E7	*Streptomyces* spp.	10	0	0	0
E8	*Streptomyces* spp.	0	0	0	0
E14	*Streptomyces* spp.	8	0	0	0
E15	*Streptomyces* spp.	0	0	0	0
H1	*Streptomyces* spp.	0	0	0	0
H2	*Streptomyces* spp.	14	0	0	0
H3	*Nocardia* spp.	0	0	0	0
H4	*Nocardia* spp.	0	0	0	0
H5	*Streptomyces* spp.	0	0	0	0
H6	*Streptomyces* spp.	20	0	0	0
H7	*Streptomyces* spp.	0	0	0	0
H11	*Streptomyces* spp.	0	0	0	0
H12	*Nocardia* spp.	11	0	0	0
H13	*Streptomyces* spp.	0	0	0	0
H15	*Nocardia* spp.	10	0	0	0
H16	*Streptomyces* spp.	26	0	0	0
H17	*Streptomyces* spp.	0	0	0	0
J1	*Streptomyces* spp.	0	0	0	0
J4	*Micromonospora* spp.	0	0	0	0
J6	*Streptomyces* spp.	15	0	0	0
J8	*Nocardia* spp.	0	0	0	0
J10	*Nocardia* spp.	10	0	0	0
J12	*Streptomyces* spp.	15	0	0	0
K1	*Streptomyces* spp.	0	0	0	0

**Table 4:** Zone of inhibition of active isolates in secondary screening.

Sample	Identified as	Test organisms			
		*S. aureus*	*E. coli*	*K. pneumoniae*	*P. aeruginosa*
A7	*Streptomyces* spp.	13	0	0	0
C1	*Streptomyces* spp.	15	15	15	0
C2	*Streptomyces* spp.	10	12	14	0
C7	*Micromonospora* spp.	17	25	18	0
C8	*Streptomyces* spp.	20	18	16	0
E4	*Nocardia* spp.	20	16	11	0
E7	*Streptomyces* spp.	15	0	0	0
E14	*Streptomyces* spp.	8	0	0	0
H15	*Nocardia* spp.	10	0	0	0
H16	*Streptomyces* spp.	10	0	0	0
J6	*Streptomyces* spp.	19	17	0	0
J10	*Nocardia* spp.	14	0	0	0
J12	*Streptomyces* spp.	14	12	0	0

## Data Availability

Further data related to this study can be made available upon request.
